# Carry-over effect of immunotherapy in patients with advanced hepatocellular carcinoma

**DOI:** 10.1007/s00262-025-04052-w

**Published:** 2025-05-16

**Authors:** Chien-Huai Chuang, Ching-Tso Chen, Chih-Hung Hsu, Yu-Yun Shao

**Affiliations:** 1 Department of Medical Oncology, National Taiwan University Cancer Center, Taipei, Taiwan; 2 Department of Oncology, National Taiwan University Hospital, 7 Chung-Shan South Road, Taipei, 10002 Taiwan; 3 Graduate Institute of Oncology, National Taiwan University College of Medicine, Taipei, Taiwan; 4 Department of Oncology, National Taiwan University Hospital Hsin-Chu Branch, Hsinchu, Taiwan; 5 Present Address: Graduate Institute of Oncology, National Taiwan University College of Medicine, Taipei, Taiwan

**Keywords:** Carry-over effect, Clinical benefit, Hepatocellular carcinoma, Immunotherapy, Prognosis, Survival

## Abstract

**Background:**

Combination immunotherapy is the current standard for treating advanced hepatocellular carcinoma (HCC). The response elicited by upfront immune checkpoint inhibitors (ICIs) might influence the efficacy of salvage therapy, a phenomenon known as the carry-over effect. This effect is thought to stem from immune memory and sustained immune activation, providing extended protection against tumor progression and resulting in a durable response even after discontinuation of ICI. This study aimed to investigate the carry-over effect of first-line ICI therapy in patients with advanced HCC.

**Methods:**

Patients who received first-line ICI therapy for advanced HCC from December 2017 to December 2021 were included if they exhibited disease progression and received second-line systemic therapy. We analyzed the associations between clinical benefit (classified as complete, partial response and stable disease) of first-line ICI therapy, post-progression survival (PPS) and second-line progression-free survival (PFS). We used a historical cohort of patients receiving first-line multikinase inhibitor (MKI) for comparison.

**Results:**

A total of 137 patients were analyzed. We included 60 patients who received first-line ICI therapy, of which clinical benefit was detected in 46 (76.7%). Compared with patients without clinical benefit of first-line ICI therapy, patients with clinical benefit exhibited significantly longer PPS (median: 14.6 vs. 4.9 months, *P* = 0.024) and second-line PFS (median: 3.6 vs. 1.6 months, *P* = 0.027). In multivariate analysis, clinical benefit of first-line ICI therapy remained an independent predictor of PPS [hazard ratio (HR): 0.295, *P* = 0.005] and second-line PFS (HR: 0.484, *P* = 0.047). Conversely, clinical benefit was not associated with PPS among patients receiving first-line MKI therapy in both univariate and multivariate analysis in historical MKI cohort.

**Conclusions:**

Clinical benefit of first-line ICI therapy was associated with PPS and second-line PFS in patients with advanced HCC, suggestive of the carry-over effect of ICI.

**Supplementary Information:**

The online version contains supplementary material available at 10.1007/s00262-025-04052-w.

## Introduction

Before the emergence of immunotherapy, multikinase inhibitors (MKIs) constituted the first-line therapy for advanced hepatocellular carcinoma (HCC). After the success of multiple clinical trials, immunotherapy has been established as the standard therapy for advanced HCC [1–3]. A combination of bevacizumab, an antiangiogenic agent, and atezolizumab, an immune checkpoint inhibitor (ICI), provided better survival benefits than sorafenib in a phase 3 clinical trial [4]. Similarly, in another phase 3 study, the overall survival (OS) was longer with a dual ICI regimen of durvalumab and tremelimumab, compared with sorafenib [5].

Compared with targeted therapy, treatment with ICIs results in a durable response because of immune memory [6]. Therefore, immunotherapy might continue providing benefits even after the treatment has been discontinued [7]—a phenomenon called the carry-over effect, which has been observed in non-small cell lung cancer, head and neck cancer, gastric cancer, and melanoma [8–11].

The carry-over effect was first described in the context of hormone-positive breast cancer, manifesting in the form of sustained advantages post cessation of endocrine therapies, such as the enduring benefits of adjuvant tamoxifen extending beyond the 5-year treatment duration [12]. However, literature is scarce on the carry-over effect of ICI therapy in the context of HCC, especially advanced or metastatic HCC.

We hypothesized that the response to first-line ICI therapy may influence post-progression clinical outcomes, suggesting a carry-over effect of immunotherapy in HCC.

## Methods

We included patients who began programmed death-1 (PD-1) blockade, alone or in combination, as first-line therapy for advanced HCC, exhibited disease progression, and received second-line systemic therapy. These patients received systemic treatment in National Taiwan University Hospital (NTUH) between December 2017 and December 2021. We reviewed medical records to retrieve the patients’ clinicopathological data. This study was approved by the Institute Research Ethical Committee of NTUH.

For comparison, we utilized data from previously published studies involving patients with advanced HCC who received MKIs as first-line therapy. These patients were enrolled in 6 clinical trials for first-line antiangiogenic targeted therapy between May 2005 and December 2010 in NTUH. This cohort was published previously [13, 14].

For study endpoints of carry-over effect, post-progression survival (PPS) was defined as the duration between the date of documented disease progression after first-line ICI therapy and death or the last follow-up visit. Progression-free survival (PFS) was defined as the duration between the start of systemic therapy and death or the date of documented disease progression. OS was defined as the duration between the start of systemic therapy and death or the last follow-up visit. Tumor response was assessed using the RECIST 1.1 guidelines, and clinical benefit was classified as complete response, partial response, or stable disease.

Statistical analyses were performed using the SAS statistical software (version 9.4, SAS Institute, Cary, NC, USA). A two-sided *P* value of ≤ 0.05 indicated statistical significance. The Kaplan–Meier method was used to estimate survival outcomes, and the log-rank test was used for intergroup comparisons. Cox’s proportional hazards model was used to adjust other potential prognostic factors in multivariate analysis.

## Results

### Patient characteristics and systemic treatment patterns

We included 60 patients, of which 6 (10.0%) were female. The median age of the cohort was 62.9 years. Hepatitis B virus surface antigen and anti-hepatitis C virus antibody were detected in the sera of 48 (80.0%) and 7 (11.7%) patients, respectively. Before first-line therapy was initiated, all patients had Child–Pugh class A liver reserve. Main portal vein thrombosis and extrahepatic metastasis were detected in 20 (33.3%) and 36 (60.0%) patients, respectively. According to the Barcelona Clinic Liver Cancer classification system, 10 patients (16.7%) were in stage B and 50 patients (83.3%) were in stage C (Table 1).

**Table 1:** Patient characteristics

	N (%)	
Total	60 (100%)	
Median age (range), in years	62.9 (23–81)	
Gender		
Female	6 (10.0%)	
Male	54 (90.0%)	
HBsAg positive	48 (80.0%)	
Anti-HCV positive	7 (11.7%)	

	Before first-line therapy	Before second-line therapy
Child–Pugh A	60 (100%)	48 (80.0%)
Main portal vein thrombosis	20 (33.3%)	29 (48.3%)
Macrovascular invasion	28 (46.7%)	35 (58.3%)
Extrahepatic spread	36 (60.0%)	40 (66.7%)
Presence of HCC in the liver	47 (78.3%)	49 (81.7%)
BCLC stage		
B	10 (16.7%)	7 (11.7%)
C	50 (83.3%)	53 (88.3%)
CLIP ≥ 3	11 (18.3%)	32 (53.3%)
AFP ≥ 400 ng/mL	24 (40.0%)	27 (45.0%)

*HBsAg* hepatitis B virus surface antigen, *HCV* hepatitis C virus, *AFP* α-fetoprotein, *BCLC* Barcelona Clinic Liver Cancer, *CLIP* Cancer of the Liver Italian Program, *HCC* hepatocellular carcinoma

The first-line ICI regimens administered were bevacizumab plus PD-1/PD-L1 blockade in 39 patients (65%), PD-1/PD-L1 blockade alone in 9 patients (15%), PD-1/PD-L1 blockade plus anticytotoxic T-lymphocyte-associated protein 4 antibody in 4 patients (6.7%), and others (PD-1/PD-L1 blockade plus novel checkpoint inhibitors) in 8 patients (13.3%). The best objective tumor responses, determined according to the RECIST 1.1, were complete response in 0 patients (0%), partial response in 12 patients (20%), and stable disease in 34 patients (56.7%). Therefore, clinical benefit was observed in 46 patients (76.7%).

When second-line therapy was initiated, 48 patients (80%) still had Child–Pugh class A liver reserve. Main portal vein thrombosis and extrahepatic metastasis were detected in 29 patients (53.3%) and 40 patients (66.7%), respectively (Table 1). The second-line therapy predominantly comprised MKIs alone (n = 49, 81.7%), such as sorafenib (n = 27, 45%), lenvatinib (n = 21, 35%), and ramucirumab (n = 1, 1.7%). The best objective tumor response to second-line therapy was complete response in 0 patients (0%), partial response in 13 patients (21.7%), and stable disease in 23 patients (38.3%). Among patients with clinical benefit of first-line ICI regimens, 30 patients (65.2%) were observed with clinical benefit of second line MKI treatment.

### Association between clinical benefit of first-line ICI therapy and PPS

**Fig. 1:**
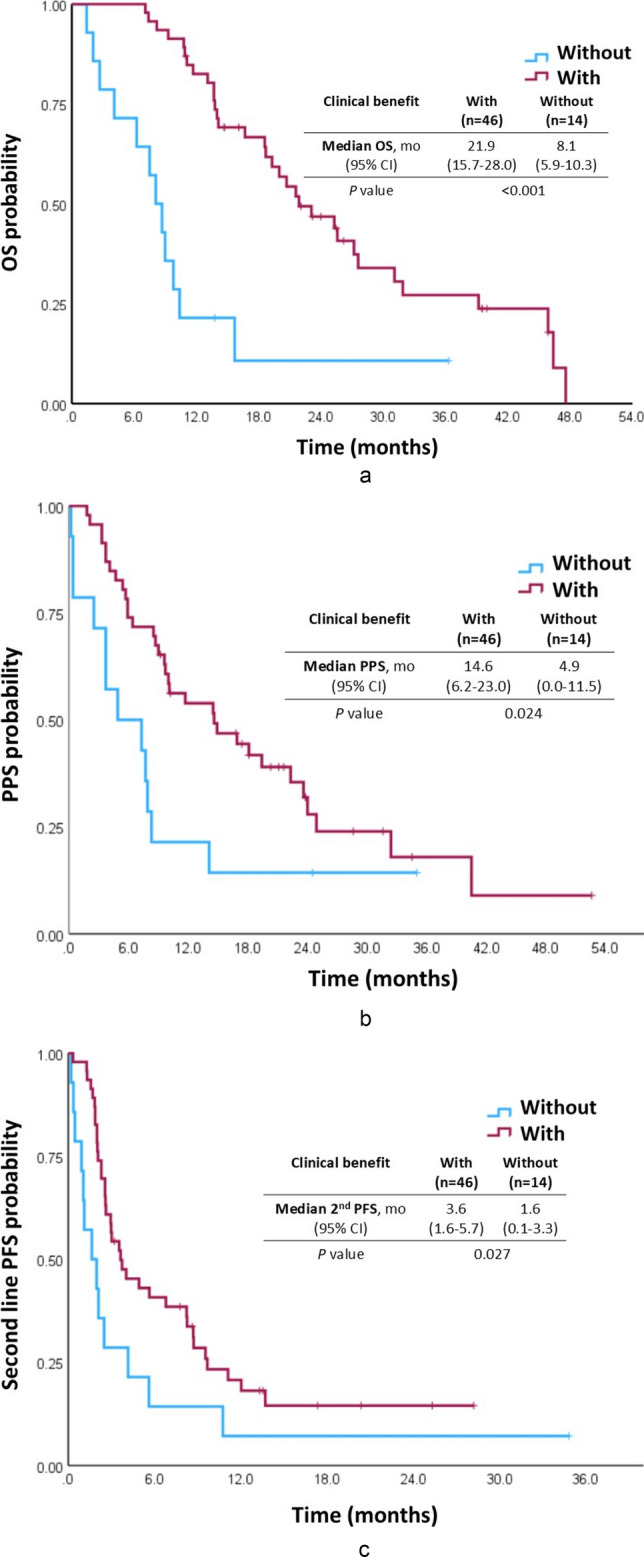
Kaplan–Meier curves of patients with HCC with and without clinical benefit of first-line ICIs. **A** Overall survival (OS), **B** post-progression survival (PPS), **C** second-line progression-free survival (PFS), grouped according to the presence or absence of clinical benefit of first-line ICI therapy; *P* values were determined using the log-rank test

The median OS of the entire cohort was 19.3 months [95% confidence interval (CI): 13.7–24.9 months]. Patients exhibiting clinical benefit to first-line ICI therapy had significantly longer OS than patients not exhibiting clinical benefit (median: 21.9 vs. 8.1 months, *P* < 0.001; Fig. 1A). The median PPS of the entire cohort was 10.0 months (95% CI: 3.8–16.2 months). Patients exhibiting clinical benefit to first-line ICI therapy had significantly longer PPS than patients not exhibiting clinical benefit (median: 14.6 vs. 4.9 months, *P* = 0.024; Fig. 1B).

In multivariate analysis, we adjusted for age, gender, hepatitis etiology, first-line ICI regimen, Cancer of the Liver Italian Program scores (including Child–Pugh score, tumor morphology, liver involvement extent, and serum alpha-fetoprotein levels), and tumor status before initiation of second-line therapy. Clinical benefit of first-line ICI therapy remained an independent predictor of longer PPS [hazard ratio (HR): 0.295, 95% CI: 0.127–0.687, *P* = 0.005; Table 2].

**Table 2:** Cox’s proportional hazards results for factors associated with PPS and second-line PFS in patients who received first-line immunotherapy

	PPS			Second-line PFS		
	HR	95% CI	*P*	HR	95% CI	*P*
Clinical benefit of first-line ICIs	0.295	0.127–0.687	0.005	0.484	0.236–0.991	0.047
ICI monotherapy (vs. combination)	0.989	0.459–2.132	0.978	1.118	0.505–2.474	0.783
Male (vs. female)	1.275	0.335–4.853	0.721	2.081	0.722–6.002	0.175
Age	1.014	0.979–1.051	0.434	1.012	0.976–1.048	0.526
HBV infection	1.094	0.360–3.322	0.874	1.151	0.395–3.357	0.797
HCV infection	0.339	0.075–1.540	0.161	0.429	0.084–2.181	0.308
CLIP ≥ 3 (vs. < 3)^a^	2.110	0.991–4.495	0.053	1.424	0.707–2.869	0.323
Macrovascular invasion^a^	3.077	0.489–19.349	0.231	1.801	0.490–6.616	0.375
Presence of HCC in the liver^a^	0.946	0.359–2.498	0.912	0.972	0.415–2.278	0.948
Extrahepatic spread^a^	0.919	0.482–1.752	0.797	0.662	0.359–1.220	0.186

*PPS* post-progression survival, *PFS* progression-free survival, *ICI* immune checkpoint inhibitor, *CLIP* Cancer of the Liver Italian Program, *HBV* hepatitis B virus, *HCV* hepatitis C virus, *HCC* hepatocellular carcinoma

^a^Before second-line therapy

### Association between clinical benefit of first-line ICI therapy and second-line PFS

The median PFS of the entire cohort during second-line systemic therapy was 3.0 months (95% CI: 1.7–4.3 months). Patients exhibiting clinical benefit to first-line ICI therapy had significantly longer second-line PPS than patients not exhibiting clinical benefit (median: 3.6 vs. 1.6 months, *P* = 0.027; Fig. 1C). In multivariate analysis, clinical benefit of first-line ICI therapy remained an independent predictor of longer second-line PFS (HR: 0.484, 95% CI: 0.236–0.991, *P* = 0.047; Table 2).

### Comparison with the MKI cohort

**Fig. 2:**
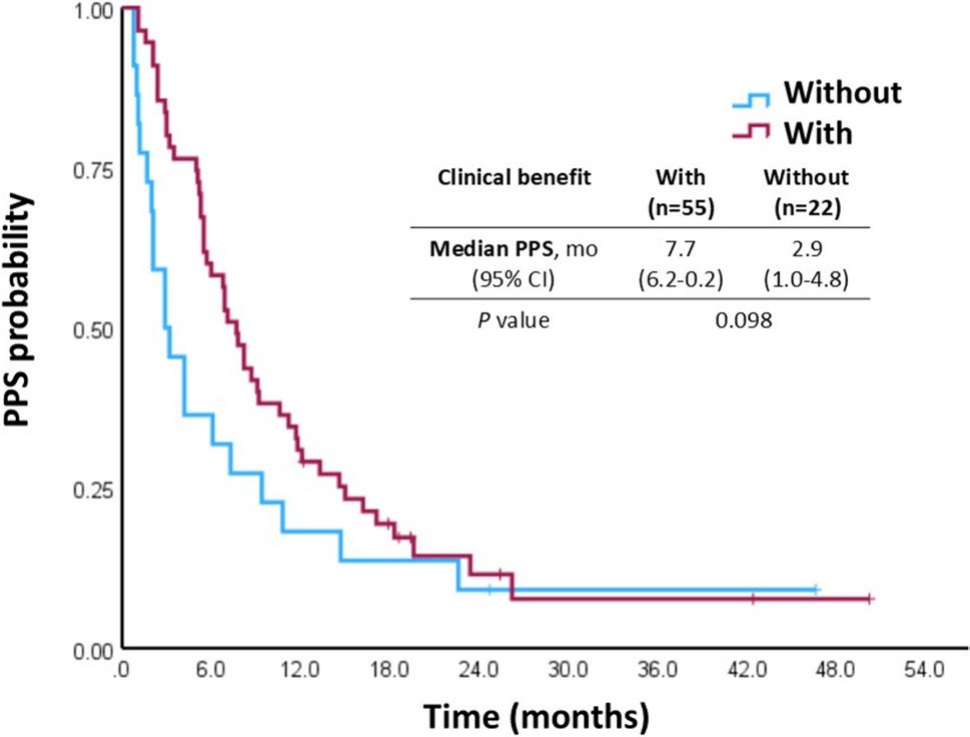
Kaplan–Meier curves for post-progression survival (PPS) in patients with and without clinical benefit from the multikinase inhibitor cohort

To discern whether the influence of clinical benefit of first-line treatment on PPS and second-line PFS was specific to ICI therapy, we utilized a historical cohort of patients enrolled in 6 clinical trials for first-line antiangiogenic targeted therapy in NTUH between May 2005 and December 2010. All 77 patients received MKIs as first-line therapy (Table S1) following a second line systemic therapy. Among second-line systemic therapy, 36 patients had received chemotherapy, and 22 patients had received thalidomide-based treatment. Clinical benefit of first-line MKI therapy was not associated with PPS in univariate analysis (median: 7.7 vs. 2.9 months, *P* = 0.098; Fig. 2) and multivariate analysis (HR: 0.581, 95% CI: 0.315–1.071, *P* = 0.082; Table 3).

**Table 3:** Cox’s proportional hazards model for factors associated with PPS in patients who received multikinase inhibitors (MKIs) as first-line therapy

	PPS		*P* value
	HR	95% CI	
Clinical benefit of first-line MKIs	0.581	0.315–1.071	0.082
Male (vs. female)	0.832	0.302–2.243	0.704
Age	1.029	1.004–1.055	0.022
HBV infection	2.060	0.913–4.650	0.082
HCV infection	0.630	0.236–1.681	0.356
CLIP ≥ 3 (vs. < 3)^a^	2.894	1.407–5.952	0.004
Macrovascular invasion^a^	1.928	0.952–3.903	0.644
Presence of HCC in the liver^a^	0.817	0.346–1.930	0.068
Extrahepatic spread^a^	3.655	1.815–7.360	< 0.001

*PPS* post-progression survival, *PFS* progression-free survival, *ICI* immune checkpoint inhibitor, *CLIP* Cancer of the Liver Italian Program, *HBV* hepatitis B virus, *HCV* hepatitis C virus, *HCC* hepatocellular carcinoma

^a^Before second-line therapy

## Discussion

In this study, we demonstrated the carry-over effect of first-line ICI therapy in patients with advanced HCC. The clinical benefit of first-line ICI therapy influenced the PPS and even the PFS of second-line systemic therapy. Such an association could not be observed for first-line MKI therapy, implying that the carry-over effect observed in our study is specific to ICI therapy. Our previous research indicated that an objective response to initial treatment with atezolizumab and bevacizumab was a predictor of survival after first-line therapy [15]. We believe this study provides novel insights into the carry-over effect in the context of HCC.

The carry-over effect of first-line ICI therapy in the context of HCC may be mediated through two mechanisms. The first mechanism involves the effects of ICI priming on the tumor microenvironment [16]. Previous study showed improved overall outcomes in patients with advanced or metastatic HCC who underwent sorafenib treatment following initial upfront anti-PD-1 therapy [17]. In preclinical model, prior anti-PD-1 therapy enhanced the tumor response to sorafenib in an orthotopic murine model of HCC. The possible explanation is anti-PD-1 therapy followed by sorafenib may exert angio-protective effects on HCC-associated blood vessels, thus amplifying the therapeutic benefits in a CD8^+^ T-cell-dependent manner and altering the tumor microenviroment [18, 19]. Additionally, immunomodulatory therapies may influence angiogenesis by stimulating immune cells that directly engage with endothelial cells [20]. A preclinical study highlighted the dynamics of a positive feedback loop between long-term vascular normalization and antitumor immunity driven by activated CD8^+^ T cells. Immune cells could induce enduring vascular normalization by interrupting angiogenic and immunosuppressive pathways, which in turn amplifies the antitumor effects of ICIs. Overall, the primary effect of ICI might enhance the treatment response, even impacting the salvage treatment efficacies resulting in carry over effect.

Another possible mechanism underlying the carry-over effect of ICI therapy is that primary resistance mechanisms against ICIs may reduce the efficacy of MKI. ICI resistance is a critical unresolved problem in HCC therapy [21]. ICI resistance can be mediated by factors that are intrinsic or extrinsic to tumor cells [22]. Budhu et al. [23]identified the molecular indicators of the immunotherapy response of patients with liver cancer. Good-survival clusters are associated with hepatocyte and liver function gene sets related to a more differentiated state, whereas poor-survival clusters (i.e., non-responders to ICIs) are associated with aggressive disease-related molecular signaling, such as epithelial cell adhesion molecule (EpCAM) signaling [23]. EpCAM is associated with increased vascularization, cancer stemness, and poor survival [24, 25]. A previous study reported that miR-181a, an oncogenic micro RNA, is overexpressed in EpCAM^+^/alpha-fetoprotein^+^ HCCs with stem cell features [26]. MiR-181 has also been demonstrated to contribute to sorafenib resistance [27]. If a patient exhibits poor response to first-line ICI therapy, tumor aggressiveness may hint at a poor response to second-line MKI therapy.

In addition to HCC, similar carry-over effects of ICI therapy have been observed in other cancer types, such as advanced clear cell renal cell carcinoma. A study reported a median PFS of 8 months associated with first-generation MKIs and 7 months associated with second-generation MKIs, with better outcomes for patients responding for a longer duration to first-line ICIs [28]. Patients with advanced clear cell renal cell carcinoma exhibited a promising response to second-line MKIs following ICIs, with a significant objective response rate over 40% and PFS of 13.2 months, which is almost similar to the historical data on the efficacy of first-line MKIs [29]. Retrospective studies on patients with melanoma, lung cancer, and head and neck cancers also revealed higher response rates following a combination therapy of ICIs and chemotherapy [8, 30, 31].

The limitations of our study primarily stem from its retrospective design and the relatively small cohort size typical of a single-center retrospective analysis. Also, the salvage treatment in the comparison cohort was not ICIs. The MKI historical cohort was not in the same timeframe as our main study cohort. Considering the overall improvement in HCC prognosis, this comparative cohort was not ideal. The limited sample size further restricted our ability to analyze the effectiveness of various combinations of first-line systemic therapies followed by different second-line treatments. To address these limitations, we employed multivariate analysis to compare and adjust for patient conditions prior to salvage treatment. While we explored the carry-over effect in the context of immunotherapy, it is important to note that the clinical benefits of first-line ICI therapy do not necessarily equate to the carry-over effect itself. Our findings suggest an association between initial treatment response and post-progression survival, but they do not establish a direct causative link. We anticipate the availability of more comprehensive and prospective data in the future owing to the increasing use of first-line ICIs in clinical practice.

In conclusion, the clinical benefit of first-line ICI therapy was associated with PPS and second-line PFS in patients with advanced HCC, which is suggestive of the carry-over effect. Such associations were not found in patients who received first-line MKI therapy. The underlying biological implications and the sequential therapeutics warrant further exploration.

## Supplementary Information

Below is the link to the electronic supplementary material.

Supplementary file1 (DOCX 20 KB)

## Data Availability

No datasets were generated or analysed during the current study.
