# Supplementary material for: Carry-over effect of immunotherapy in patients with advanced hepatocellular carcinoma
Source: Cancer Immunol Immunother. 2025 May 16;74(7):208. doi: 10.1007/s00262-025-04052-w (PMC12084203; doi:10.1007/s00262-025-04052-w)
Supplement: Supplementary file 1 — Supplementary file1 (DOCX 20 KB) [file 262_2025_4052_MOESM1_ESM.docx]

**Table S1** Characteristics of patients from the historical multikinase inhibitor cohort

|  | N (%) | |
| --- | --- | --- |
| Total | 77 (100%) | |
| Median age (range), in years | 56.8 (23-83) | |
| Gender | | |
| Female | 7 (10%) | |
| Male | 70 (90%) | |
| HBsAg positive | 61 (79.2%) | |
| Anti-HCV positive | 9 (11.7%) | |
|  | Before first line therapy | Before second line therapy |
| Child-Pugh A | 77 (100%) | 66 (85.7%) |
| Main portal vein thrombosis | 17 (22.1%) | 23 (30.0%) |
| Macrovascular invasion | 38 (49.4%) | 44 (57.1%) |
| Extrahepatic spread | 50 (65.0%) | 56 (72.7%) |
| Presence of HCC in the liver | 63 (81.8%) | 65 (84.4%) |
| BCLC stage | | |
| B | 10 (13.0%) | 10 (13.0%) |
| C | 67 (87.0%) | 67 (87.0%) |
| CLIP ≥3 | 22 (28.6%) | 33 (42.9%) |
| AFP ≥400 ng/mL | 34 (44.2%) | 36 (46.8%) |

Abbreviations: HBsAg = hepatitis B virus surface antigen; HCV = hepatitis C virus; AFP = α-fetoprotein; BCLC = Barcelona Clinic Liver Cancer; CLIP = Cancer of the Liver Italian Program; HCC = hepatocellular carcinoma.

| Table S2 Characteristics of patients who received first-line immunotherapy before second-line therapy | | | | |
| --- | --- | --- | --- | --- |
|  | Clinical benefit of first-line ICI (N=46) | | Non-clinical benefit of first-line ICI (N=14) |  |
|  | **N (%)** | | | *P* |
| Child-Pugh A | 40 (87.0%) | | 8 (57.1%) | 0.039 |
| Main portal vein thrombosis | 20 (43.5%) | | 9 (64.3%) | 0.290 |
| Macrovascular invasion | 23 (50.0%) | | 12 (85.7%) | 0.028 |
| Extrahepatic spread | 31 (67.4%) | | 9 (64.3%) | 1.000 |
| Presence of HCC in the liver | 36 (78.3%) | | 13 (92.3%) | 0.430 |
| BCLC stage |  |  |  |  |
| B | 2 (4.3%) | | 5 (35.7%) | 0.006 |
| C | 44 (95.7%) | | 9 (64.3%) | 0.006 |
| CLIP ≥3 | 21 (45.7%) | | 11 (78.6%) | 0.037 |
| AFP ≥400 ng/mL | 16 (34.8%) | | 11 (78.6%) | 0.006 |

Abbreviations: ICI = immune checkpoint inhibitor; AFP = α-fetoprotein; BCLC = Barcelona Clinic Liver Cancer; CLIP = Cancer of the Liver Italian Program; HCC = hepatocellular carcinoma.
